# *Amburana cearensis* leaf extract protects against cisplatin-induced ovarian damage through regulation of p-PTEN and p-Akt proteins in mice

**DOI:** 10.22038/IJBMS.2022.58927.13092

**Published:** 2022-06

**Authors:** Bruna B. Gouveia, Ricássio S. Barberino, Vanúzia G. Menezes, Alane P. O. Monte, Regina Lucia S. Silva, Raimundo C. Palheta Jr, Larissa A. Rolim, Emanuella C. V. Pereira, Raimundo G. Oliveira Jr, Jackson Roberto G. S. Almeida, Maria Helena T. Matos

**Affiliations:** 1 Nucleus of Biotechnology Applied to Ovarian Follicle Development, Department of Veterinary Medicine, Federal University of São Francisco Valley, Petrolina, Pernambuco, Brazil; 2 Laboratory of Veterinary Pharmacology, Department of Veterinary Medicine, Federal University of São Francisco Valley, Petrolina, Pernambuco, Brazil; 3 Center for Drug, Medicament and Food Analysis, Department of Pharmacy, Federal University of São Francisco Valley, Petrolina, Pernambuco, Brazil; 4 Center for Studies and Research of Medicinal Plants, Department of Pharmacy, Federal University of São Francisco Valley, Petrolina, Pernambuco, Brazil

**Keywords:** Anti-oxidants, Antineoplastic protocols, Fertility preservation, Ovarian follicle, Phytotherapy

## Abstract

**Objective(s):**

To evaluate the effects of *Amburana cearensis* leaf extract against cisplatin-induced ovarian toxicity in mice and involvement of p-PTEN and p-Akt proteins.

**Materials and Methods:**

*A. cearensis* ethanolic leaf extract was analyzed by high-performance liquid chromatography (HPLC). Mice were pretreated once daily for 3 days as follows: (1) the control group was pretreated with oral administration (o.p.) of saline solution, followed by intraperitoneal (IP) injection of saline solution. The other groups were pretreated (o.p.) with (2) saline solution (cisplatin group), (3) *N-*acetylcysteine (positive control), with (4) 50, or (5) 200 mg/kg body weight of *A. cearensis* extract, followed by injection of 5 mg/kg body weight (IP) of cisplatin. The ovaries were harvested and destined for histological (follicular morphology), immunohistochemistry (apoptosis and cell proliferation), and fluorescence (reactive oxygen species [ROS], glutathione concentrations [GSH], and active mitochondria) analyses. Furthermore, immunoexpression of p-PTEN and p-Akt was evaluated to elucidate a potential mechanism by which *A. cearensis* extract could prevent cisplatin-induced ovarian damage.

**Results:**

After HPLC analysis, protocatechuic acid was detected in the extract. The pretreatment with *N*-acetylcysteine or *A. cearensis* extract maintained the percentage of normal follicles and cell proliferation, reduced apoptosis and ROS concentrations, and increased GSH concentrations and mitochondrial activity compared with cisplatin treatment. Furthermore, pretreatment with *A. cearensis* extract regulated p-PTEN and p-Akt immunoexpression after cisplatin exposure.

**Conclusion:**

Pretreatment with *A. cearensis* extract prevented cisplatin-induced ovarian damage through its anti-oxidant actions and by modulating the expression of phosphorylated PTEN and Akt proteins.

## Introduction

*Amburana cearensis* (Allemão) A.C. Smith is a native species from the Caatinga biome, commonly found in the semiarid region of Northeastern Brazil. Different parts of the plant have been used in folk medicine for treatment of headaches, muscle aches, constipation, urinary tract infections, and gynecological inflammation because of their antinociceptive, anti-inflammatory, and anti-oxidant properties (1, 2). The anti-oxidant effect of *A. cearensis* is attributed to the secondary metabolites identified in the extract, such as gallic acid, protocatechuic acid, epicatechin, *p*-coumaric acid, and kaempferol (3), which induced a decrease in the reactive oxygen species (ROS) production, and/or an increase in the expression of enzymatic and non-enzymatic anti-oxidants, thus preventing cellular damage from oxidative stress (4, 5). This anti-oxidant activity may be crucial during cancer treatment in female patients since the gonadotoxic impact of chemotherapeutic agents is mainly through the oxidative stress generated in the ovary (6, 7). 

Ovarian toxicity induced by chemotherapy may be associated with the use of platinum compounds, such as cisplatin (*cis*-diaminodichloroplatinum), which increased ROS production and decreased cellular anti-oxidant capacity, resulting in mitochondrial and DNA damages and consequently follicle cell apoptosis (8, 9). Follicular loss induced by cisplatin is related to the death of oocytes in primordial follicles with the involvement of proteins such as phosphatase and tensin homolog deleted on chromosome 10 (PTEN) and protein kinase B (Akt) (10). However, there are no reports on the effects of *A. cearensis* extract on cisplatin-induced ovarian toxicity and its potential mechanisms of action (possible involvement of p-PTEN and p-Akt proteins) in the ovary.

Because the use of herbal products and extracts is common throughout the world (11), and to aid local exploitation of herbal medicines, the present study aimed to evaluate whether pretreatment with *A. cearensis* extract could prevent cisplatin-induced ovarian damage in a mouse model, and to investigate if p-PTEN and p-Akt proteins are involved in the protective effect of this extract.

## Materials and Methods

***Chemicals***

Cisplatin was obtained from Libbs Farmacêutica Ltda (São Paulo, Brazil). *N*-acetylcysteine and phosphate-buffered saline (PBS) were obtained from Sigma Aldrich Chemical Co (St. Louis, USA). Saline solution, 10% buffered formalin, ethanol, paraffin, citrate buffer, and H_2_O_2_ were obtained from Dinâmica (São Paulo, Brazil). The rabbit polyclonal antibodies anti-PCNA (proliferating cell nuclear antigen), anti-activated caspase-3, anti-p-PTEN (Ser 380), and anti-p-Akt (Ser 473) were obtained from Santa Cruz Biotechnology (CA, USA). Decloaking chamber, 1% normal goat serum, MACH4 Universal HRP-polymer, and diaminobenzidine were purchased from Biocare (Concord, USA). The fluorescent markers 2’, 7’-dichlorodihydrofluorescein diacetate (H2DCFDA) and CellTracker® Blue (CMF2HC) were obtained from Invitrogen Corporation (Carlsbad, USA), and Mitotracker® Red (CMXRos) from Molecular Probes (Melbourne, Australia). Companies of other chemicals were mentioned in the text. 

***Plant material and extract preparation***

Fresh leaves from wild *A. cearensis* were collected in Petrolina (09°2305500 South and 38°–40°3000300 West, Pernambuco, Brazil). All procedures for access to genetic patrimony and associated traditional knowledge were carried out and the project was registered in SisGen (n° A0585A9). Dried and pulverized leaves (1,296 g) were submitted to exhaustive maceration using ethanol 95% as solvent. Successive extractions were performed with solvent renewal every 72 hr. The extractive solution was filtrated and concentrated on a rotatory evaporator at a maximum temperature of 50 °C, resulting in the ethanol extract (106.5 g, yield 12.38%), which was stored at 4 °C until its use. The extract was diluted in saline solution, corresponding to doses of 50 or 200 mg/kg.

***Analysis of ethanolic extract by high-performance liquid chromatography (HPLC)***

Chromatographic equipment consisted of a Shimadzu^®^ liquid chromatograph equipped with an autosampler (SIL-20 A) and a diode array detector (SPD-M20A). The data were acquired and processed using Shimadzu^®^ LC solution 1.0 software (Japan). The stationary phase used was a C-18 column Agilent^®^ (250 x 4.6 mm, 5 µm). As mobile phase, two solutions were used: solution A, water + 0.1% trifluoroacetic acid and solution B, acetonitrile, following the gradient: 0–40min (0-40% B), 40–50 min (40% B), 50–60 min (40-0% B). The temperature was kept constant at 30 °C, the injection volume was 10 µl for hydroalcoholic fluid extract (1 mg/ml), crude ethanolic extract (1 mg/ml), and for standard solutions (200 µg/ml) under a flow rate of 0.8 ml/min. The analyzes were made at a wavelength of 340 nm (12).

The following phenolic compounds were analyzed for qualitative determination: caffeic acid, chlorogenic acid, *p*-coumaric acid, protocatechuic acid, tannic acid, gallic acid, catechin, canferol, galocatequin, myricetin, quercetin, isoquercetin, resveratrol, rutin, scopoletin, harman, hesperetin, hesperidine, at concentrations of 100 and 200 µg/ml. To identify the presence of these compounds in the sample, the retention time and UV spectrum (λ_max_) of the standard substance chromatograms were compared with the corresponding peaks present in the sample chromatogram.

***Animals and experimental design***

Experimental protocols were performed according to the Ethics Committee on Animal Use, Federal University of São Francisco Valley (protocol number 0005/230518). Adult female Swiss mice (n = 25; age: 8 weeks; weight: 30–45 g) were housed at a temperature of 25 ºC and kept under a 12/12 hr light/dark cycle. Animals were provided free access to standard chow and water. 

Mice were randomly divided into five experimental groups (5 animals per group) and pretreated once daily for 3 days as follows: (1) the control group was pretreated orally by gavage (p.o.) with saline solution (0.15 M, 0.3 ml/mouse) and 1 hr later received an intraperitoneal injection (IP) of saline solution (0.15 M, 0.15 ml/mouse). The other groups were pretreated (p.o.) with (2) saline solution (0.15 M, 0.3 ml/mouse; cisplatin group), (3) *N-*acetylcysteine (150 mg/kg body weight; positive control), or with (4) 50 or (5) 200 mg/kg body weight of *A. cearensis* leaf extract. After 1 hr of oral administration, mice from groups 2 to 5 received cisplatin (5 mg/kg body weight, IP). Those 3 days after initial treatment with a total dose of 15 mg/kg cisplatin-induced ovarian damage as previously described (8,13). The administered doses of *A. cearensis* extract (4) and *N-*acetylcysteine (8, 13) were based on previous studies where there were indications of their protective effects. Mice were euthanized on the fourth day, and ovarian tissues were used for histological, immunohistochemical, and fluorescence analyses. 

***Histology and follicle counting***

The ovaries (one ovary per animal) were fixed in 10% buffered formalin overnight and embedded in paraffin. The blocks were sectioned at 5 μm, stained with hematoxylin-eosin (Vetec, São Paulo, Brazil) and then examined using a light microscope (Nikon, Tokyo, Japan). Follicles were classified using morphological criteria previously described by Pedersen and Peters (14) in primordial (one layer of flattened or flattened and cuboidal granulosa cells), primary (a complete layer of cuboidal granulosa cells surrounding the oocyte), secondary (two or more layers of cuboidal granulosa cells and no sign of antrum formation), and antral (multiple granulosa cell layers with some antral space). In addition, follicles were classified as morphologically normal if no clear signs of degeneration were noted, which included shrunken oocytes, disorganization of the granulosa cell layer, condensed nuclear chromatin, and/or cell swelling.

To evaluate follicular activation, only morphologically normal follicles with a visible oocyte nucleus were counted, and the proportion of primordial and growing (primary, secondary, and antral) follicles was calculated in the different treatments. Overall, 150 follicles were evaluated for each treatment (30 follicles per treatment × five replicates = 150 follicles) according to Barberino *et al*. (8). All counts were performed blindly.

***Immunohistochemistry***

Sections (5 μm thick) from each paraffin block were subjected to antigen retrieval with citrate buffer at 95 °C in a decloaking chamber for 40 min, and endogenous peroxidase activity was prevented by incubation with 3% H_2_O_2_ and methyl ethanol (QEEL, São Paulo, Brazil) for 10 min. Nonspecific binding sites were blocked using 1% normal goat serum in PBS. Subsequently, the sections were incubated in a humidified chamber for 90 min at room temperature with rabbit polyclonal anti-PCNA (1:100) and anti-activated caspase-3 (1:100). Thereafter, the sections were incubated for 30 min with MACH4 Universal HRP-polymer. Protein localization was demonstrated with diaminobenzidine, and the sections were counterstained with hematoxylin (Vetec) for 1 min. For reaction control, the tissues were incubated with blocking buffer, without the antibody included. The number of PCNA-positive granulosa and theca cells (brown staining) was counted in 10 random fields per treatment. For evaluation of apoptosis (immunoexpression of activated caspase-3), 40–45 follicles were evaluated in each treatment, and those containing oocytes and/or 70% of granulosa cells stained in brown were considered apoptotic.

We also evaluated the immunoexpression of p-PTEN and p-Akt in the ovaries of mice of the control and those treated with cisplatin combined with 50 mg/kg *A. cearensis* extract or not (lower responsive dose that produced the best protective results). Immunohistochemistry was performed using the antibodies anti-p-PTEN (1:100) and anti-p-Akt (1:75), and then immunostaining was subjectively classified as absent, weak, moderate, or strong in the follicular cells. All immunohistochemical analyzes were performed according to a previous study (13) by an experienced investigator.

***Fluorescence microscopy***

Preantral and antral follicles (n = 40 per treatment) were mechanically isolated using 26-gauge needles from the ovaries (one ovary per animal) and incubated in the dark with 10 μM H_2_DCFDA, 10 μM CellTracker Blue, and 100 nM Mitotracker Red to detect the concentrations of ROS, GSH, and active mitochondria as green, red and blue, respectively. Follicles were observed under an epifluorescence microscope (Nikon) with UV filters (460 nm for ROS, 370 nm for GSH, and 579 nm for active mitochondria). The fluorescence intensities were analyzed using the Image J software (Version 1.41; National Institute of Health, Bethesda, MD, USA) and normalized to the follicles of the control group (13). 

***Statistical analysis***

Data regarding normal, primordial, and growing (activation) follicles, PCNA-positive cells, and apoptotic follicles were compared using the Chi-square test and expressed as percentages. Data from ROS, GSH, and active mitochondria were evaluated using the Shapiro-Wilk test to verify the normal distribution of residuals and homogeneity of variances. Thereafter, the Kruskal-Wallis nonparametric test was used for comparisons. When the main effects or interactions were significant, means were compared using Student Newmans Keuls. The results were expressed as means ± standard error of mean (SEM), and all tests were processed in the BioEstat 4.0 software considering P<0.05 as significant.

## Results

***Analysis of the A. cearensis extract through the HPLC method***

**Figure 1:**
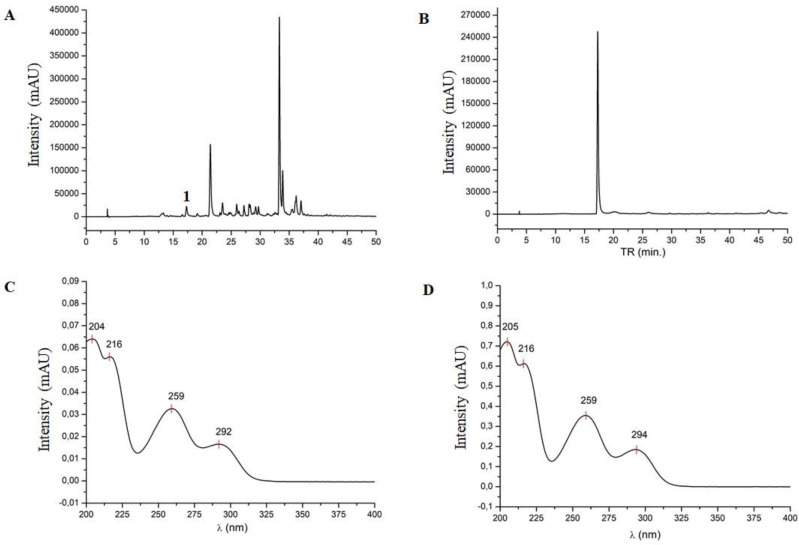
Chromatogram of *Amburana cearensis* ethanolic extract obtained by HPLC (A; “1" indicates the peak referring to protocatechuic acid) and protocatechuic acid standard (B) at 270 nm. UV spectrum of protocatechuic acid in the sample (C) and the protocatechuic acid standard (D)

After analysis of the ethanolic extract through the HPLC method, it was possible to obtain the chromatographic profile of the sample and identify only the presence of protocatechuic acid (peak 1 in Figure 1A) in the retention time of 17.29 min (λ = 204/216/259/292). The chromatogram of the protocatechuic acid standard is shown in Figure 1B, and the UV spectra of the protocatechuic acid in the sample and the standard are shown in Figures 1C and 1D, respectively. It was not possible to identify the major compounds observed at retention times of 21.5, 33.3, and 33.9 min.

***Effect of A. cearensis extract on follicular morphology and primordial follicle activation***

**Figure 2:**
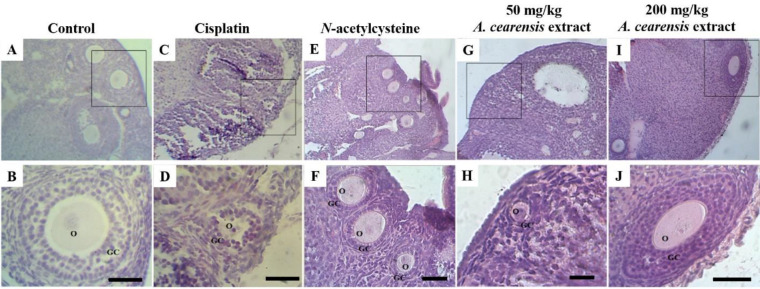
Histological sections of mouse ovarian fragments in the control group (A, B), exposed to cisplatin alone (C, D), and exposed to *N*-acetylcysteine (E, F), 50 (G, H) or 200 (I, J) mg/kg *Amburana cearensis* extract plus cisplatin. O: oocyte; GC: granulosa cells; Scale bars: 50 µm (A, C, E, G and I: 100x; B, D, F, H and J: 400x)

Figure 2 demonstrates that ovaries from mice of the control group showed normal follicular morphology with centrally located oocytes and granulosa cells surrounded by normal intact basement membranes (Figures 2A and 2B). The cisplatin group had damaged ovarian structure and showed follicles with swollen and disorganized granulosa cells, and retracted or vacuolated oocytes (Figures 2C and 2D). Pretreatment with *N-*acetylcysteine (Figures 2E and 2F) and 50 (Figures 2G and 2H) or 200 (Figures 2I and 2J) mg/kg *A. cearensis* extract decreased the follicular damage caused by cisplatin, and the follicles showed morphological features similar to those of the control group.

**Figure 3:**
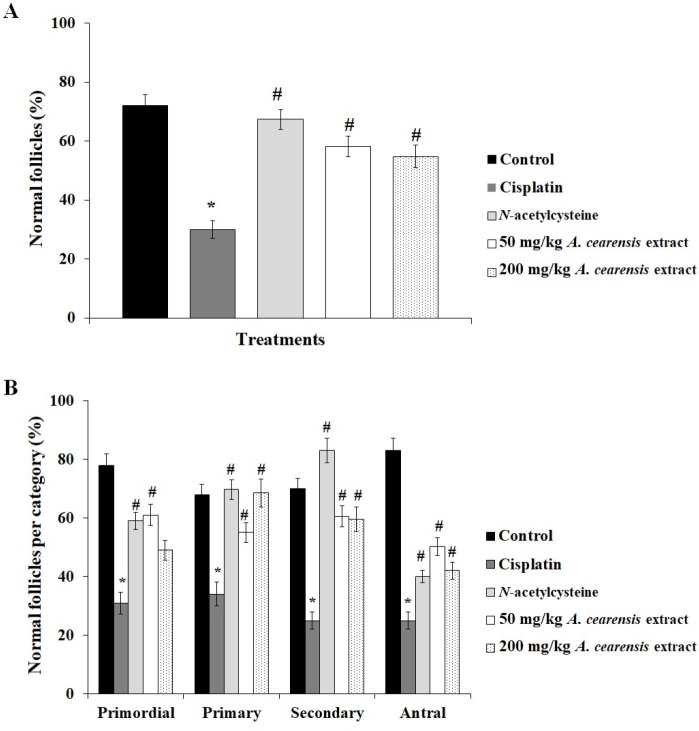
Total follicular survival (percentage of morphologically normal follicles; A), and percentage of morphologically normal follicles in different stages (primordial, primary, and secondary follicles; B) in the control group, mice exposed to cisplatin alone, and exposed to *N-*acetylcysteine or A. cearensis extract (50 or 200 mg/kg) before cisplatin treatment. *Cisplatin compared with the control group; #*N-*acetylcysteine or *Amburana cearensis* extract pretreatment compared with cisplatin only group (*P*<0.05)

Cisplatin treatment decreased (*P*<0.05) the percentage of morphologically normal follicles (30%) in comparison with the control group (72.12%). *N-*acetylcysteine (67.33%) and *A. cearensis* extract (50 mg/kg: 58.1% or 200 mg/kg: 54.82%) pretreatment prevented (*P*<0.05) reduction in the percentage of normal follicles induced by cisplatin (Figure 3A). Regarding the different developmental stages, cisplatin treatment decreased (*P*<0.05) the percentage of normal primordial, primary, and secondary follicles, compared with the control (Figure 3B). Cisplatin-induced changes were reversed by pretreatment with *N-*acetylcysteine and 50 mg/kg *A. cearensis* (*P*<0.05). Noteworthy, 200 mg/kg *A. cearensis* extract showed percentages of normal primordial follicles similar (*P*>0.05) to the cisplatin group. There were no differences (*P*>0.05) in the percentages of antral follicles between the groups probably due to the sample size. Primordial follicle activation was not observed in any treatment (*P*>0.05).

***Effect of A. cearensis extract on cell proliferation and apoptosis***

**Figure 4:**
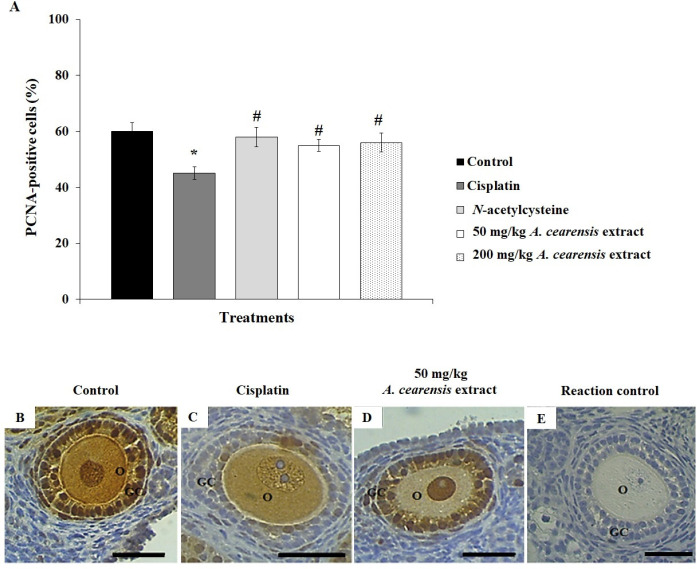
Percentage of PCNA-positive cells (A) in the control group, mice exposed to cisplatin alone, and exposed to *N*-acetylcysteine or *Amburana cearensis* extract (50 or 200 mg/kg) before cisplatin treatment. *Cisplatin compared with the control group; #N-acetylcysteine or *A. cearensis *extract pretreatment compared with cisplatin only group (*P*<0.05). Immunohistochemical localization of PCNA-positive cells: follicles in the control group (B), treated with cisplatin alone (C), or pretreated with 50 mg/kg *A. cearensis* extract before cisplatin treatment (D). Reaction control (E). O: oocyte; GC: granulosa cells; Scale bars: 50 µm (400x)

**Figure 5:**
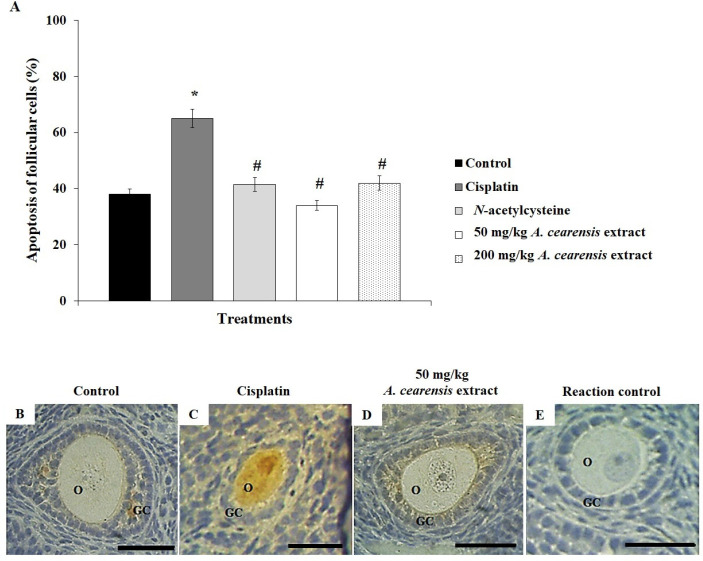
Percentage of apoptosis of follicular cells (A) in the control group mice exposed to cisplatin alone and exposed to *N*-acetylcysteine or *Amburana cearensis* extract (50 or 200 mg/kg) before cisplatin treatment. *Cisplatin compared with the control group; #*N*-acetylcysteine or *A. cearensis* extract pretreatment compared with cisplatin only group (*P*<0.05). Immunohistochemical localization of activated caspase-3: follicles in the control group (B), treated with cisplatin alone (C), or pretreated with 50 mg/kg* A. cearensis* extract before cisplatin treatment (D). Reaction control (E). O: oocyte; GC: granulosa cells; Scale bars: 50 µm (400x)

Cisplatin treatment decreased (*P*<0.05) the percentage of PCNA-positive cells (Figure 4A) and increased apoptosis (Figure 5A) compared with the control. Pretreatment with N-acetylcysteine and 50 or 200 mg/kg *A. cearensis* extract prevented (*P*<0.05) reduction in cell proliferation (Figure 4A-D) and decreased (*P*<0.05) the apoptosis (Figure 5A-D) induced by cisplatin. Reaction control is shown in Figures 4E and 5E.

***Effect of A. cearensis extract on concentrations of ROS, GSH, and active mitochondria***

**Figure 6:**
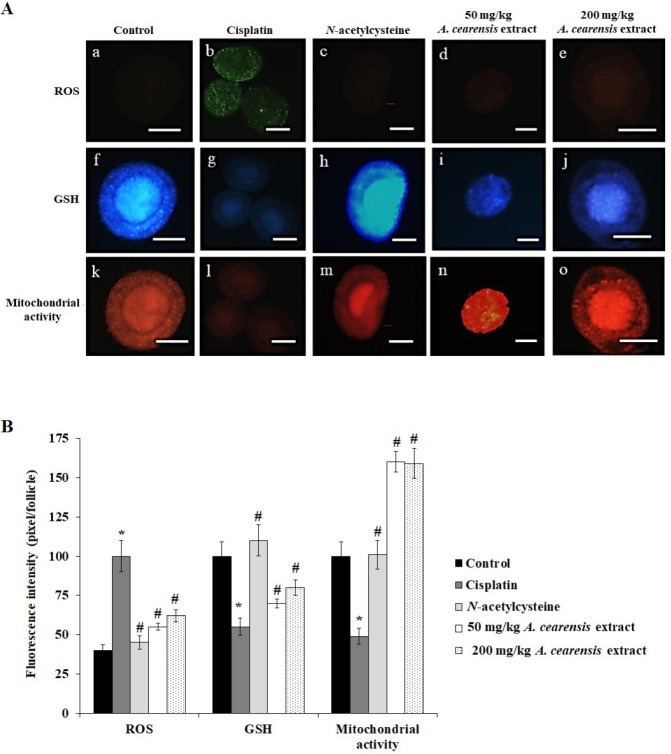
Detection of intracellular concentrations of reactive oxygen species (ROS), glutathione concentrations (GSH), and mitochondrial activity (A): follicles in the control (a, f, k), treated with cisplatin alone (b, g, l), pretreated with *N*-acetylcysteine (c, h, m), and pretreated with 50 (d, i, n) or 200 (e, j, o) mg/kg *Amburana cearensis* extract before cisplatin treatment. Scale bars: 50 μm (100x). Intracellular concentrations of ROS, GSH, and mitochondrial activity (B) in follicles of different groups exposed to cisplatin alone or in association with *N*-acetylcysteine, 50 or 200 mg/kg *A. cearensis* extract. *Cisplatin compared with the control group; #*N*-acetylcysteine or *A. cearensis* extract pretreatment compared with cisplatin only group (*P*<0.05)

Figure 6 shows that ROS concentrations were greater, while GSH concentrations and mitochondrial activity were lower in the cisplatin group compared with the control (*P*<0.05). Cisplatin-induced changes were reversed by *N-*acetylcysteine and *A. cearensis* extract (50 or 200 mg/kg) pretreatment in all fluorescent markers evaluated (*P*<0.05).

***Effect of A. cearensis extract on p-PTEN and p-Akt immunoexpression***

**Figure 7:**
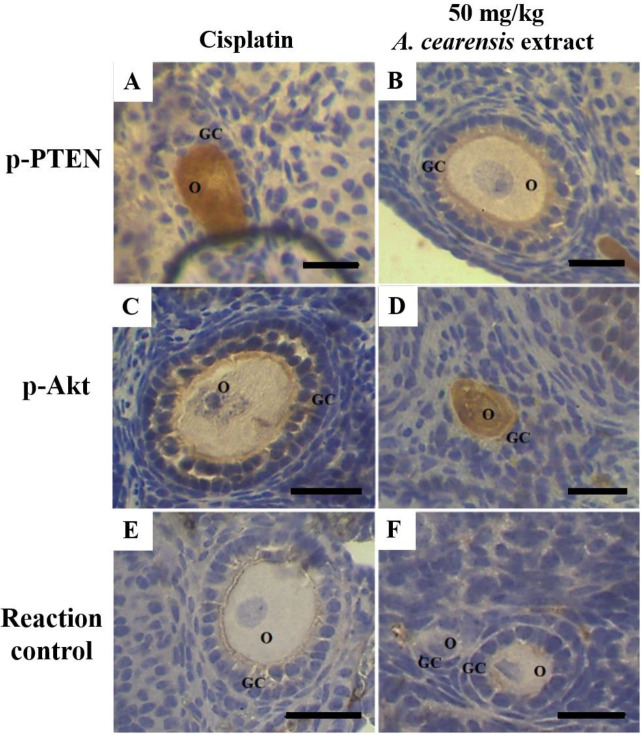
Immunohistochemical localization for p-PTEN (A, B) and p-Akt (C, D): follicles exposed to cisplatin alone (A, C) or exposed to 50 mg/kg A. cearensis extract before cisplatin treatment (B, D). Reaction controls (E, F). O: oocyte; GC: granulosa cells; Scale bars: 50 µm (400x)

Follicles from the cisplatin group showed a moderate to strong staining for p-PTEN (Figure 7A), where as pretreatment with 50 mg/kg *A. cearensis* extract attenuated (weak staining) this cisplatin-induced PTEN phosphorylation (Figure 7B). The p-PTEN immunoexpression in the control group was similar to the *A. cearensis* extract group (weak). Furthermore, weak staining for p-Akt in follicles from the cisplatin group (Figure 7C), while moderate staining for p-Akt was observed in follicles from mice in the control group and pretreated with *A. cearensis* extract (Figure 7D). Reaction control did not show any background staining (Figures 7E and 7F).

## Discussion

The present study demonstrated that pretreatment with *A. cearensis* leaf extract before cisplatin administration showed more normal follicles and less apoptosis, besides increased cell proliferation and GSH concentrations and decreased ROS production and mitochondrial injury compared with cisplatin treatment alone. A previous study demonstrated that oral administration of *A. cearensis* stem bark extract daily for up to 50 days showed minimal biochemical alterations and did not show any teratogenicity in rats (15), indicating that *A. cearensis* extract is non-toxic and well-tolerated. Furthermore, the extract of *A. cearensis* exhibited neuroprotective potential against oxidative stress induced by glutamate (4) and maintained survival, and stimulated the *in vitro* growth of goat secondary follicles by improving GSH concentration and mitochondrial activity (16). 

In the present study, it was possible to identify protocatechuic acid in the *A. cearensis* leaf extract, which is an endogenous plant phenol with anti-oxidant action (17). Although other peaks have been observed in the chromatographic profile of the sample, they do not correspond to any of the phenolic compounds that were used for qualitative determination. Phytochemical characterization of different *A. cearensis* parts (stem bark, seeds, and leaves) has been performed using different analytical techniques (1). To our knowledge, only one study has analyzed the phytochemical profile of *A. cearensis* leaf ethanolic extract through the HPLC method and the authors identified protocatechuic acid, epicatechin, p-coumaric acid, gallic acid, and kaempferol (3). These differences in the chemical composition of the extracts may be related to the temperature, relative humidity, solar radiation, and soil composition in which the plant grows (18).

Protocatechuic acid protected mouse ovarian follicles (13) and rat kidney cells (19) against cisplatin-induced toxicity, reduced apoptosis, and stimulated the development of ovine secondary follicles cultured *in vitro* (20). Furthermore, other phenolic compounds (epicatechin, p-coumaric acid, gallic acid, and kaempferol) that may be present in the *A. cearensis* leaf extract also demonstrated protective effects against cisplatin-induced toxicity in rodent organs (kidney: 21; testis: 22; ovary: 23). Taken together, these findings provide evidence that the natural anti-oxidants of *A. cearensis* extract, especially protocatechuic acid, may have helped to reduce the ovarian damage caused by cisplatin.

In the present study, *N-*acetylcysteine, a GSH precursor that acted as a positive control, and both 50 and 200 mg/kg *A. cearensis* extracts showed equal results in most of the evaluated endpoints. Thus, to elucidate a possible molecular mechanism of protection of *A. cearensis* extract against cisplatin-induced ovarian damage, we evaluated the immunoexpression of the phosphorylated forms of PTEN and Akt proteins in the ovaries from mice of the control and those treated with cisplatin combined or not with 50 mg/kg *A. cearensis* extract. In this study, treatment with cisplatin resulted in increased p-PTEN and reduced p-Akt expression. Although some authors have shown that cisplatin promoted follicular activation via the PI3K/PTEN/Akt pathway (24), this was not observed in our study. Our findings are consistent with a previous study that demonstrated that short-term treatment with 5 mg/kg cisplatin or continuous injections with 2 mg/kg cisplatin once daily for 15 days induces the oocyte death of primordial follicles without follicular activation (10). Nevertheless, the present study showed that pretreatment with 50 mg/kg *A. cearensis* extract decreased p-PTEN and enhanced p-Akt expression when compared with the cisplatin group. Akt is phosphorylated at Ser-473 residue by the mammalian target of rapamycin complex 2 (mTORC2) whose activity maintains follicular survival (25). Protocatechuic acid, identified in the extract, has already been shown to increase the expression of p-Akt (Ser 473) in liver cells of diabetic rats, improving cellular metabolism and protecting against oxidative stress (26). Furthermore, reduced p-PTEN expression is involved in the protective role of the protocatechuic acid against cisplatin-induced ovarian damage (13). Thus, it is likely that treatment with 50 mg/kg *A. cearensis* reduced apoptosis and maintained normal cell proliferation and follicle development during cisplatin treatment by regulating p-PTEN and p-Akt proteins.

## Conclusion

*A. cearensis* extract at 50 mg/kg protected mouse ovaries during cisplatin experimental chemotherapy, maintaining follicular morphology, inhibiting apoptosis and mitochondrial damage, and promoting cell proliferation through its anti-oxidant effects (reducing ROS production and stimulating GSH expression) and by regulating immunoexpression of p-PTEN and p-Akt proteins. Therefore, understanding the actions of *A. cearensis* extract in the ovary can aid local exploitation of herbal medicine. However, future studies should be done to evaluate the effect of other doses of *A. cearensis* extract or the effects of long-term treatment, as well as to assess whether pretreatment with *A. cearensis* extract does not interfere with the effectiveness of cancer treatment.

## Authors’ Contributions

BBG, RCPJr, and MHTM Conceived the study and design; ECVP and RGO Processed plant material; BBG, RSB, VGM, and APOM Performed experiments; BBG Analyzed and interpreted results; BBG, RSB, VGM, and APOM Prepared the draft manuscript; RCP, LAR, JRGSA, and MHTM Critically revised the paper; MHTM Supervised the research; BBG, RSB, VGM, APOM, RCP, LAR, ECVP, RGO, JRGSA, and MHTM Approved the final version to be published.

## Conflicts of Interest

None of the authors has any conflicts of interest to declare.
